# 

**Published:** 1891-06

**Authors:** 


					﻿Vol. XI.
JUNE, 1891.
NO. 6.
IBHE
HOMEOPATHIC pHY^lClAJfl,
A Monthly Journal of Homoeopathic Materia Medica
and Clinical Medicine.
“He is a freeman whom truth makes free, and all are slaves beside."
“Seek the Truth: come whence it may, cost what it will."
EDITED AND PUBLISHED BY
WALTER M. JAMES, M. D.,
AND
George H. Clark, m. D.
PHILADELPHIA :
No. 1125 8FRTTCK STREET.
LONDON
ALFRED HEATH & CO., Sole Agents for Great Britain,
114 Ebury Street, Eaton Square.
AS“Fearly Subscription, $2.50. invariably in advance. Single numbers, 25 cts.
Entered at the Philadelphia Poet-OfBee as Second-Claw Mail Matter.
				

